# Arrhythmogenic Right Ventricle in Left Ventricular Non-compaction - In response to "Right Ventricular Ablation as a Therapeutic Option for Left Ventricular Hypertrabeculation / noncompaction"

**DOI:** 10.1016/s0972-6292(16)30740-9

**Published:** 2014-03-12

**Authors:** Shohreh Honarbakhsh, Irina Suman-Horduna, Lilian Mantziari, Sabine Ernst

**Affiliations:** 1 Royal Brompton and Harefield NHS Foundation Trust, United Kingdom; 2 NIHR Cardiovascular Biomedical Research Unit, Royal Brompton and National Heart and Lung Institute, Imperial College London

**Keywords:** Right Ventricular Tachycardia, Left Ventricular Non-compaction Cardiomyopathy

 We welcome Dr Finsterer's and Dr Stollberger's interest in our manuscript and their insightful observations. We have recently reported [1] a case of successful mapping and ablation of ventricular tachyarrhythmia in a patient with left ventricular non-compaction (LVNC). The arrhythmia proved to have a rather unusual and unexpected origin, located within a structurally normal right ventricle (RV). Our patient was followed up for one year following his procedure and had no recurrence of symptoms, documented ventricular ectopy (VE) or ventricular tachycardia on Holter monitoring.

 It is noteworthy that the first procedure that our patient underwent was done in another centre and the VEs at that time were mapped and ablated at the RV outflow tract (RVOT). In the second procedure the VEs were mapped at the base of the RV towards the infero-lateral aspect of the tricuspid annulus indicating a different arrhythmia focus. The first procedure was done one year prior to the second presentation with arrhythmias, and no RVOT arrhythmias were documented spontaneously or induced at the time of the second ablation procedure. The appearance of the RV free wall in our patient was normal with no discernible pathology in the targeted area on steady-state free precession cines or late gadolinium cardiac magnetic resonance images. Thereby, in this case, two different arrhythmia foci were identified from a structurally normal RV.

 Our patient had undergone a 2D echocardiography prior to his first ablation that had demonstrated increased trabeculation of the LV suggestive of a diagnosis of LVNC. It was not until he was referred to our centre for his second procedure that he underwent CMR, which confirmed this diagnosis. It is well established that CMR is superior to 2D echocardiography in diagnosis of LVNC [2,3].

 We appreciate that LVNC is a genetically heterogeneous disorder with a sporadic and familial form [4,5]. In the case illustrated in our report, there was no known family history of either diagnosed LVNC, symptoms of palpitations or documented arrhythmias, suggesting that he had a sporadic variant of this condition. In a study by Oechslin et al. out of 34 adult patients with LVNC, in only 18% of the patients the disease was familial [5], while in study by Ritter et al. [3] a familial occurrence of only 12% was reported, emphasizing that a sporadic variant of LVNC is common.

 As Drs Finsterer and Stollberger highlighted, LVNC may be linked to mutations in mitochondrial, cytoskeletal, Z-line and sarcomeric proteins [6]. Our patient did not undergo genetic testing and this was mainly because he had no children or siblings that were at risk of this condition. Secondly, as underlined by Dr Finsterer and Stollberger, genetic testing has indicated important genetic heterogeneity [7], as well as a lack of specific genotype-phenotype association; therefore, we believe that knowing the genetic mutation does not provide additional information on the possible severity of the cardiomyopathy the patient has, and it is not part of routine investigation usually carried out to confirm this diagnosis.

 It is well recognized that patients with LVNC may be asymptomatic, present with congestive heart failure, ventricular arrhythmias or systemic emboli [4,8]. Our patient had no previous history of stroke or systemic embolism, however this would not exclude a diagnosis of LVNC. In the largest prospective cohort study of LVNC patients, only 24% had thromboembolic events [5]. There are no clinical guidelines to suggest that cerebral MRI should be used to diagnose cerebral events in an asymptomatic patient.

 We fully agree that there are still outstanding questions regarding LVNC. Through our case we have illustrated one face of a larger spectrum of clinical manifestations in this condition, and demonstrated that patients with LVNC may have an arrhythmogenic substrate that can extend beyond the LV; furthermore, this focus can be mapped and ablated successfully.

## References

[R1] Honarbakhsh S (2013). Successful Right Ventricular Tachycardia Ablation in a Patient with Left Ventricular Non-compaction Cardiomyopathy. Indian Pacing Electrophysiol J.

[R2] Weiss F (2003). MRI in the diagnosis of non-compacted ventricular myocardium (NCVM) compared to echocardiography. Rofo Fortschr Geb Rontgenstr Neuen Bildgeb Verfahr.

[R3] Thuny F (2010). Assessment of left ventricular noncompaction in adults: side-by-side comparison of cardiac magnetic resonance imaging with echocardiography. Arch Cardiovasc Dis.

[R4] Ritter M (1997). Isolated noncompaction of the myocardium in adults. Mayo Clin Proc.

[R5] Oechslin EN (2000). Long-term follow-up of 34 adults with isolated left ventricular noncompaction: a distinct cardiomyopathy with poor prognosis. J Am Coll Cardiol.

[R6] Sen-Chowdhry S (2008). Left ventricular noncompaction and cardiomyopathy: cause, contributor, or epiphenomenon. Curr Opin Cardiol.

[R7] Oechslin E (2011). Left ventricular non-compaction revisited: a distinct phenotype with genetic heterogeneity?. Eur Heart J.

[R8] Chin TK (1990). Isolated noncompaction of left ventricular myocardium. A study of eight cases. Circulation.

